# Systematic gene expression analysis of putative target genes linked to miR-31 in 83 oral squamous cell carcinoma samples

**DOI:** 10.1186/s13005-024-00443-z

**Published:** 2025-02-25

**Authors:** Carolin Feldges, Susanne Jung, Nikolai Purcz, Christoph Sproll, Johannes Kleinheinz, Sonja Sielker

**Affiliations:** 1 Vascular Biology of Oral Structures (VABOS) Research Unit; Department of Cranio-Maxillofacial Surgery, University Hospital Muenster, Waldeyerstraße 30, Muenster, 48149 Germany; 2 Department of Cranio-Maxillofacial Surgery, University Hospital Kiel, Kiel, Germany; 3 Department of Cranio-Maxillofacial Surgery, University Hospital Duesseldorf, Duesseldorf, Germany

**Keywords:** Oral squamous cell carcinoma, MiR31, Target genes, Oncogenes, Tumour suppressors, Angiogenesis

## Abstract

**Background:**

The development of oral squamous cell carcinoma on the molecular level and the resulting prognosis for patients have remained poorly understood. While AngiomiR-31 was implicated in the progression and metastasis of OSCC. However, this connection has not yet been investigated in more detail and tested for its significance with regard to new therapies and the prognosis of patients.

**Methods:**

Through a systemic analysis of putative target genes of AngiomiR-31 in OSCC, this study aimed to highlight possible prognostic markers and genes that might improve prognostic predictability in patients with oral squamous cell carcinoma, especially regarding AngiomiR-31 as an outstanding mediator of angiogenesis. The study is based on gene data from 83 OSCC samples. Potentially relevant genes were selected and sorted by TNM, grading and UICC in these 83 OSCC whole-genome microarray datasets. Data was analysed and tested for significance.

**Results:**

Through our investigation 20 potential target genes, including tumor suppressor genes, oncogenes and genes not yet categorized, were found and their expression correlated significantly with the expression of AngiomiR-31.

**Conclusion:**

These findings contribute to a more profound understanding of the molecular mechanisms underlying OSCC progression and may have implications for the development of novel therapeutic strategies targeting AngiomiR-31 in OSCC. Further validation of these genes is needed to validate their clinical relevance and potential as prognostic markers or therapeutic targets in OSCC.

## Background

Oral squamous cell carcinoma (OSCC) is a relevant type of carcinoma, and 2%−4% of all cancer cases worldwide are oral carcinomas. Therefore, OSCC is the most common malignant epithelial neoplasm affecting the oral cavity. The increasing number of cases in younger, lighter-skinned individuals and the relatively late diagnosis of these carcinomas are also important for an advanced understanding of this type of carcinoma [1].

MicroRNAs are single-stranded RNAs (approximately 22 nucleotides in length) involved in gene expression regulation and consequently in the expression of oncogenes, tumour suppressors and metastasis regulators. “They downregulate the expression of genes encoding proteins or long noncoding RNAs (lncRNAs) by inhibiting mRNA translation or by promoting target RNA degradation” [2]. They are also responsible for balancing differentiation, proliferation, and cell death [3]. Through these functions, tumour progression can be influenced by microRNAs. MicroRNAs have various putative targets, and the expression of microRNAs in tissues is sometimes extremely diverse [4]. It has already been confirmed that microRNA-31 (miR-31; AngiomiR-31) has various molecular functions. These include binding to the 3'UTR end during translation. Regulatory sequences at the 3'UTR can therefore influence translation in various ways and ultimately regulate the expression of multiple proteins and genes [2, 5, 6].

The formation of new blood vessels, also known as angiogenesis, plays a key role in tumour development through supplying the tumour with nutrients and draining off degradation products, thus influencing the progression and growth of the tumour. Tumours form complex vascular networks to continuously ensure this supply stability. In addition to growth factors and vascular genes, which control angiogenesis, microRNAs are involved in angiogenesis [4, 7]. These are also referred to as AngiomiRs. Various AngiomiRs have been shown to influence angiogenesis in head and neck tumours. Among others, these include miR-21 [8]. In another paper on lingual SCC, a significant correlation between miR-21 and tumour cell apoptosis was found. This finding suggested that angiomiR-21 is an independent prognostic marker in these tumours [9]. A 2022 meta-analysis evaluated results from 10 studies related to miR-21 expression and overall survival and revealed a clear correlation between these two factors, indicating that miR-21 is a prognostic factor [10]. It was found that growth factors that influence angiogenesis also influence the expression of miR-31 [11]. Previous scientific work by our group revealed significant overexpression of miR-31 in oral SCC and a significant correlation between miR-31 expression and tumour grade [8]. The detailed microRNA 31 expression data related to tumour grade were as follows: G1 = 0.75; G2 = 13.6; and G3 = 16.6. (p value = 0.003). In general, miR-31 was highly expressed. In contrast, other miRNAs, such as miR-21, showed no correlation with clinical parameters [12]. To summarize, microRNAs in general or miR-31 have the potential to be utilized and manipulated therapeutically to disrupt angiogenesis and, consequently, the supply of tumours, thereby halting or slowing their growth. MicroRNAs can even be detected in saliva in the context of oral diseases [13]. However, current OSCC research lacks a comprehensive analysis of multiple genes from a sample pool that are associated with promising microRNAs and are relevant to cancer cell migration and invasion.

This underlines how important it is to investigate their effects, expression and target genes because these findings could ultimately lead to more convenient early detection methods for OSCC. This study provides insight into the particular relationship between miR-31 and tumour progression, but further research is needed regarding the expression of miR-31 in tumour samples and saliva. The expression of miR-31 is influenced by various target genes. We searched various databases for possible target genes of human miR-31 and then examined the expression profiles of 115 of these target genes. In general, we investigated various results in the field of tumour research, looking for genes that might serve as prognostic markers to verify them and to determine which genes are influenced by microRNAs. For example, it has already been shown in other publications that *MLNA* is a possible prognostic marker [14]. In the next step, we analysed the gene expression pattern of *MLNA* in our panel of whole genome data from 83 OSCC samples. The aim was to identify target genes that correlate with our tumour parameters significantly. Potential prognostic markers and therapeutic targets were identified. These studies are based on whole-genome miRNA microarray data and not on individual expression data obtained via real-time qPCR. This approach should simplify further scientific research and processing and serve as good comparison with other results on this topic. Overall, these findings could enable clinicians to better predict the prognosis of patients with oral squamous cell carcinoma.

## Methods

### Patient data

Patient data was described previously [8, 12, 15]. In brief, 83 OSCC tissue samples were taken during tumour surgery after informed consent was obtained from patients between 2009 and 2012. Data collection included patients who were histologically diagnosed with OSCC. Patients were over 18 years old and had not yet received any adjuvant radiation or chemotherapy. One patient with a recurrent tumour (T1 tumour) was included. In the control group, healthy tissue samples (*n* = 30) were taken from the oral vestibular mucosa during orthognathic or traumatologic surgery after providing informed consent. The tissue samples were snap-frozen in liquid nitrogen after surgery and stored at −80 °C until further use. The ethics committee of the medical faculty approved the study; the ID of ethical clearance (WWU Muenster) is 2008–580-f-s, and the study is registered in a public Clinical Trials Registry, DRKS00000199.

The resulting data pool is part of previous studies [8, 12, 15].

### RNA extraction and microarray assay

The entire study design, including the RNA extraction, microarray assay, and bioinformatic steps, has been described in detail previously [8, 12]. Brief, total RNA, including miRNA, was isolated with the miRNeasy Mini Kit (Qiagen, Hilden, Germany). Gene and miRNA expression analysis was performed with the Whole Human Gene Expression Microarray (4 × 44 K; GPL4133) and with the human miRNA microarray (V2; GPL8936; based on Sanger miRBase release 10.1) according to the manufacturer’s protocol. Microarrays were scanned using an Agilent G2505B Microarray Scanner, feature extraction was performed with Feature Extraction software version 9.5, and data was analysed with GeneSpring GX 7.3.1. The first normalization step consisted of background elimination, while during the second step, the 50th percentile of each spot was normalized. Normalization to the healthy oral mucosa pool was performed in the last step by setting the expression factor for the healthy oral mucosa pool to 1. Primary statistical analysis was performed with GeneSpring GX 7.3.1 software. The microarrays used in this study, scanner, and analysis software were obtained from Agilent Technologies (all Agilent Technologies, Waldbronn, Germany).

### Identification of putative target genes of miR-31

A literature search was carried out for potential target genes related to miR-31 with search keys (miR-31 in OSCC, HNSCC or cancer). Furthermore, a database search was performed within the DIANA database (http://diana.imis.athena-innovation.gr/DianaTools/index.php). A total of 253 potential target genes were identified. These genes were matched and sorted with the gene expression data provided by the microarray analysis. The first step was to identify genes that were not present in our expression data. In the second step, genes were sorted by mean gene expression, evaluated and statistically analysed. Finally, genes were subdivided into three groups: known tumour suppressor genes (*n* = 11), known oncogenes (*n* = 25) and not precisely assignable genes (*n* = 9).

### Statistical analysis

The statistical analysis was performed using the statistical software SPSS version 28 (IBM, Ehningen, Germany), and the computations used were previously described [15]. Due to the non-normal and non homogeneous distribution of the data, the Kruskal‒Wallis test and ANOVA were used. The level of significance was set at *p* < 0.05. The parameters used were UICC classification, T-status, lymph node status, tumour grade, smoking status, alcohol abuse status and type of OSCC.

## Results and discussion

Eighty-three OSCC samples were collected between 2009 and 2012. The study was previously published in parts, and detailed information about the study design and patient data was described [8, 12, 15]. Whole-genome and whole-miRnome analyses were concurrently performed, and mRNA and microRNA analyses were performed on the same sample preparation. The expression profiles of 83 OSCC samples and a pool of healthy mucosa samples (*n* = 30) were used as controls. Table 1 gives an overview of the clinicopathological features of the included OSCC patients.

**Table 1:** An overview of the clinicopathological features included in the present study of OSCC patients [8, 12, 15]

**Group**		**Number**	**(%)**
T-status^a^	T1 + T2	56	(67)
	T3 + T4	27	(33)
G-status	G1	2	(2.4)
	G2	67	(80.7)
	G3	14	(16.9)
N-status	N-	53	(64)
	N +	30	(36)
UICC classification (TNM 7th edition; 2009)	UICC1	15	(18.1)
	UICC2	23	(27.7)
	UICC3	8	(9.6)
	UICC4	37	(44.6)
Type of OSCC	keratinized	67	(81)
	not-keratinized	9	(11)
	n/a	7	(8)
Smoker	Yes	49	(59)
	No	31	(37)
	n/a	3	(4)
Alcohol abusus	Yes	48	(58)
	No	32	(39)
	n/a	3	(4)
Smoker & alcohol		40	(48)
Localization	mouth floor	23	(27.7)
	alveolar ridge	22	(25.4)
	tongue	20	(24)
	buccal plain	8	(9.6)
	lip	5	(6)
	palate	3	(3.6)

% Percentage of sample size

*n/a* data not avaible

^a^one recurrent tumour in T1 group

In a previous study, a significant correlation between miR-31 expression and tumour grade was found [12]. The detailed microRNA 31 expression data in relation to tumour grade were as follows: G1 = 0.75; G2 = 13.6; and G3 = 16.6 (*p* value = 0.003). In addition, microRNA 31 was generally highly expressed. In contrast, other microRNAs, such as microRNA 21, showed no correlation with clinical parameters [12]. Therefore, we started a literature search and a database search of potential target genes of miR-31. A total of 253 potential target genes were identified. The next step involved generating a gene expression profile based on the whole-genome array data. Subsequently, genes that lacked information or were not included in the complete microarray data were excluded. Additional genes that exhibited irregular or mixed expression were excluded. At least, genes which expression did not differ from that of miR-31 were excluded. We identified 11 known tumour suppressor genes, 25 known oncogenes, and 9 genes that have not yet been precisely assigned. Tables 2, 3 and 4 summarize the fold changes in the expression of these genes according to T status, tumour grade, lymph node status, and UICC classification. The percentage of tumour samples exhibiting up- or downregulated genes or miR-31 is presented. Only two samples exist for grade group G1. The expression rate of some genes varied between the two samples; both expression patterns are depicted. These results reveal a broad spectrum of interesting genes in terms of their impact on disease progression and prognosis, especially for OSCC associated with miR-31.

**Table 2:** Expression fold changes of putative target genes of miR-31, known as tumour suppressor genes

**Gene**	**All**	**T1 + T2**	**T3 + T4**	**G1**^**a**^	**G2**	**G3**	**N0**	**N + **	**UICC 1**	**UICC 2**	**UICC 3**	**UICC 4**
	*n* = 83	*n* = 56	*n* = 27	*n* = 2	*n* = 68	*n* = 13	*n* = 53	*n* = 30	*n* = 16	*n* = 23	*n* = 8	*n* = 36
hsa-miR-31	10.77 ± 11.96 80%	10.08 ± 11.83 80%	12.39 ± 12.29 81%	0.36 ± 0.12 100%	10.13 ± 12.16 81%	14.12 ± 10.81 92%	10.45 ± 12.33 85%	11.66 ± 11.4 72%	6.43 ± 5.98 70%	11.12 ± 14.43 95%	9.09 ± 10.71 75%	12.69 ± 11.97 78%
*PCDH9*	0.18	0.21	0.13	0.42	0.19	0.12	3.73	0.18	0.27	0.20	0.17	0.16
NM_020403	± 0.18	± 0.21	± 0.09	± 0.11	± 0.19	± 0.17	± 0.21	± 0.15	± 0.24	± 0.23	± 0.12	± 0.13
	95%	93%	100%	100%	96%	92%	96%	93%	93%	96%	100%	95%
*Meis*	0.24	0.28	0.17	0.22//1.2	0.23	0.33	1.52	0.19	0.34	0.31	0.17	0.18
NM_002398	± 0.18	± 0.20	± 0.06		± 0.15	± 0.28	± 0.20	± 0.10	± 0.25	± 0.20	± 0.08	± 0.09
	98%	96%	100%	50%//50%	100%	92%	96%	100%	93%	96%	100%	100%
*CNOT4*	0.67	0.65	0.72	0.88	0.67	0.71	1.18	0.74	0.59	0.68	0.65	0.72
NM_001008225	± 0.15	± 0.15	± 0.15	± 0.21	± 0.15	± 0.14	± 0.15	± 0.13	± 0.15	± 0.16	± 0.11	± 0.15
	73%	71%	78%	100%	74%	72%	80%	63%	80%	78%	88%	65%
*GRHL1*	0.46	0.41	0.55	0.59	0.46	0.42	1.63	0.41	0.54	0.43	0.44	0.46
NM_198182	± 0.26	± 0.25	± 0.27	± 0.16	± 0.27	± 0.21	± 0.26	± 0.25	± 0.30	± 0.22	± 0.32	± 0.25
	84%	82%	89%	100%	84%	85%	77%	97%	80%	74%	100%	89%
*NFIA*	0.34	0.37	0.28	0.24	0.34	0.32	0.37	0.3	0.49	0.35	0.3	0.29
NM_005595	± 0.16	± 0.17	± 0.11	± 0.4	± 0.15	± 0.15	± 0.17	± 0.09	± 0.21	± 0.14	± 0.1	± 0.11
	99%	98%	100%	100%	99%	100%	100%	100%	100%	100%	100%	97%
*PLCB1*	0.46	0.47	0.43	0.61//2.41	0.46	0.46	1.41	0.50	0.55	0.39	0.46	0.49
NM_015192	± 0.24	± 0.25	± 0.21		± 0.23	± 0.28	± 0.24	± 0.23	± 0.24	± 0.25	± 0.28	± 0.22
	77%	77%	78%	50%//50%	74%	100%	74%	86%	60%	83%	75%	81%
*DIRAS*	0.35	0.36	0.34	0.69	0.35	0.3	1.33	0.37	0.53	0.27	0.27	0..38
NM_004675	± 0.23	± 0.23	± 0.24	± 0.34	± 0.23	± 0.28	± 0.24	± 0.23	± 0.31	± 0.12	± 0.15	± 0.25
	89%	86%	100%	100%	91%	77%	87%	93%	80%	91%	75%	95%
*AIM1*	0.53	0.5	0.58	0.7	0.52	0.58	1.30	0.48	0.44	0.62	0.43	0.54
NM_001624	± 0.22	± 0.22	± 0.21	± 0.21	± 0.21	± 0.26	± 0.20	± 0.25	± 0.23	± 0.13	± 0.22	± 0.25
	81%	80%	81%	100%	79%	92%	77%	90%	87%	74%	88%	81%
*ALDH5A1*	0.45	0.42	0.5	0.70	0.43	0.46	1.64	0.46	0.56	0.35	0.5	0.47
NM_170740	± 0.24	± 0.24	± 0.23	± 0.11	± 0.25	± 0.25	± 0.25	± 0.23	± 0.25	± 0.22	± 0.25	± 0.24
	79%	77%	81%	100%	78%	92%	74%	87%	64%	83%	100%	78%
*SPTBN1*	0.43	0.45	0.41	0.54	0.44	0.41	1.2	0.45	0.52	0.41	0.29	0.46
NM_003128	± 0.18	± 0.19	± 0.16	± 0.06	± 0.18	± 0.2	± 0.17	± 0.19	± 0.22	± 0.14	± 0.1	± 0.19
	98%	96%	100%	100%	97%	100%	96%	100%	87%	100%	100%	100%
*DLC1*	0.53	0.55	0.48	0.69//1.51	0.55	0.44	1.3	0.49	0.61	0.57	0.41	0.52
NM_182643	± 0.23	± 0.23	± 0.23		± 0.22	± 0.29	± 0.24	± 0.18	± 0.1	± 0.29	± 0.17	± 0.23
	75%	70%	85%	50%//50%	74%	92%	75%	60%	67%	78%	63%	78%

± standard deviation; % percent of samples in group, *T* T-status, *G* grading, *N* lymph nodes, *UICC* UICC classification

^a^group G1 includes two samples, the results are less robust;//both results in group G1 are shown

**Table 3:** Expression fold changes of putative target genes for miR-31 known as tumour oncogenes

**Gene**	**All**	**T1 + T2**	**T3 + T4**	**G1**^**a**^	**G2**	**G3**	**N0**	**N + **	**UICC 1**	**UICC 2**	**UICC 3**	**UICC 4**
	*n* = 83	*n* = 56	*n* = 27	*n* = 2	*n* = 68	*n* = 13	*n* = 53	*n* = 30	*n* = 16	*n* = 23	*n* = 8	*n* = 36
hsa-miR-31	10.77 ± 11.96 80%	10.08 ± 11.83 80%	12.39 ± 12.29 81%	0.36 ± 0.12 100%	10.13 ± 12.16 81%	14.12 ± 10.81 92%	10.45 ± 12.33 85%	11.66 ± 11.4 72%	6.43 ± 5.98 70%	11.12 ± 14.43 95%	9.09 ± 10.71 75%	12.69 ± 11.97 78%
*SELK*	0.59	0.60	0.55	1.14	0.59	0.57	1.23	0.53	0.72	0.62	0.54	0.55
NM_021237	± 0.15	± 0.16	± 0.14	± 0.10	± 0.16	± 0.13	± 0.15	± 0.14	± 0.18	± 0.15	± 0.13	± 0.13
	93%	91%	96%	100%	94%	100%	92%	93%	70%	100%	100%	95%
*CMYA5*	0.28	0.31	0.23	0.27//1.25	0.29	0.27	15.84	6.29	0.34	0.32	0.3	0.25
NM_153610	± 0.22	± 0.21	± 0.20		± 0.24	± 0.41	± 0.15	± 0.30	± 0.28	± 0.31	± 0.43	± 0.27
	78%	77%	81%	50%//50%	79%	77%	80%	77%	70%	70%	75%	81%
*C1QTNF7*	0.10	0.13	0.05	0.15	0.11	0.04	1.19	0.11	0.2	0.07	0.11	0.09
NM_031911	± 0.13	± 0.15	± 0.04	± 0.02	± 0.14	± 0.03	± 0.12	± 0.15	± 0.17	± 0.07	± 0.11	± 0.13
	98%	96%	100%	100%	99%	92%	96%	100%	93%	96%	100%	100%
*KIAA0232*	0.54	0.54	0.54	0.61	0.54	0.52	1.66	0.52	0.61	0.55	0.42	0.53
NM_014743	± 0.16	± 0.16	± 0.14	± 0.03	± 0.16	± 0.17	± 0.15	± 0.17	± 0.13	± 0.16	± 0.16	± 0.16
	88%	88%	89%	100%	90%	77%	91%	83%	87%	96%	88%	84%
*USP47*	0.47	0.47	0.48	0.37	0.47	0.48	1.69	0.45	0.54	0.45	0.61	0.43
NM_017944	± 0.17	± 0.17	± 0.16	± 0.26	± 0.16	± 0.22	± 0.17	± 0.16	± 0.15	± 0.17	± 0.2	± 0.14
	92%	88%	100%	100%	96%	72%	92%	90%	87%	91%	100%	92%
*KCNC1*	0.3	0.31	0.27	0.18//1.04	0.31	0.27	3.10	0.31	0.41	0.27	0.32	0.28
NM_004976	± 0.18	± 0.19	± 0.16		± 0.17	± 0.22	± 0.17	± 0.2	± 0.23	± 0.15	± 0.17	± 0.18
	88%	84%	96%	50%//50%	90%	92%	85%	93%	70%	83%	100%	95%
*NEBL*	0.35	0.34	0.38	0.62	0.33	0.43	1.36	0.32	0.4	0.34	0.32	0.35
NM_006393	± 0.22	± 0.22	± 0.22	± 0.06	± 0.21	± 0.19	± 0.23	± 0.19	± 0.29	± 0.17	± 0.23	± 0.21
	94%	89%	100%	100%	94%	92%	89%	97%	87%	91%	100%	97%
*OGT*	0.67	0.66	0.70	1.11	0.67	0.71	1.09	0.67	0.68	0.67	0.67	0.69
NM_003605	± 0.15	± 0.14	± 0.16	± 0.10	± 0.14	± 0.17	± 0.16	± 0.12	± 0.14	± 0.16	± 0.13	± 0.15
	76%	77%	74%	100%	78%	77%	85%	60%	70%	91%	100%	68%
*AS3MT*	0.42	0.45	0.36	0.33	0.42	0.36	3.21	0.42	0.50	0.45	0.38	0.37
NM_020682	± 0.20	± 0.22	± 0.13		± 0.21	± 0.21	± 0.2	± 0.22	± 0.23	± 0.20	± 0.27	± 0.18
	80%	77%	85%	100%	82%	72%	81%	79%	80%	83%	57%	84%
*C1orf24*	0.24	0.25	0.21	0.22	0.25	0.18	0.26	0.21	0.32	0.27	0.18	0.21
NM_052966	± 0.15	± 0.17	± 0.10	± 0.29	± 0.15	± 0.09	± 0.17	± 0.11	± 0.20	± 0.18	± 0.07	± 0.11
	100%	100%	100%	100%	100%	100%	100%	100%	100%	100%	100%	100%
*PDE5A*	0.30	0.31	0.29	0.64	0.3	0.33	1.77	0.31	0.38	0.28	0.26	0.31
NM_001083	± 0.19	± 0.2	± 0.19	± 0.13	± 0.19	± 0.19	± 0.2	± 0.19	± 0.25	± 0.16	± 0.18	± 0.2
	95%	93%	100%	100%	96%	92%	96%	93%	87%	100%	100%	95%
*KCNIP2*	0.4	0.41	0.38	0.51	0.37	0.51	1.78	0.41	0.44	0.41	0.27	0.41
NM_173342	± 0.16	± 0.17	± 0.15	± 0.11	± 0.15	± 0.18	± 0.15	± 0.18	± 0.17	± 0.16	± 0.13	± 0.17
	92%	88%	100%	100%	90%	100%	87%	100%	67%	91%	100%	100%
*ZC3H6*	0.37	0.37	0.36	0.38	0.35	0.39	1.68	0.35	0.47	0.34	0.39	0.34
NM_198581.3	± 0.19	± 0.19	± 0.18	± 0.36	± 0.18	± 0.2	± 0.21	± 0.13	± 0.24	± 0.19	± 0.23	± 0.14
	96%	95%	100%	100%	97%	92%	96%	97%	93%	96%	100%	97%
*COL9A3*	0.25	0.26	0.23	0.23//1.84	0.25	0.25	3.66	0.21	0.35	0.25	0.3	0.20
NM_001853	± 0.19	± 0.21	± 0.14		± 0.19	± 0.19	± 0.22	± 0.11	± 0.23	± 0.24	± 0.22	± 0.1
	96%	96%	96%	50%//50%	99%	92%	94%	100%	87%	100%	100%	97%
*WASF3*	0.44	0.45	0.34	0.86	0.44	0.38	1.43	0.43	0.57	0.46	0.42	0.4
NM_006646	± 0.24	± 0,25	± 0.2	± 0.05	± 0.24	± 0.26	± 0.25	± 0.24	± 0.29	± 0.26	± 0.17	± 0.23
	90%	88%	96%	100%	90%	92%	89%	93%	87%	87%	100%	92%
*EPM2AIP1*	0.56	0.57	0.54	1.00	0.55	0.6	1.15	0.54	0.71	0.55	0.58	0.54
NM_014805	± 0.17	± 0.18	± 0.15	± 0.13	± 0.16	± 0.25	± 0.18	± 0.16	± 0.2	± 0.16	± 0.20	± 0.15
	88%	86%	93%	100%	91%	92%	87%	90%	70%	91%	75%	95%
*FAM46A*	0.22	0.22	0.21	0.2//3.52	0.23	0.17	2.52	0.18	0.19	0.27	0.17	0.21
NM_017633	± 0.15	± 0.15	± 0.16		± 0.16	± 0.08	± 0.17	± 0.11	± 0.10	± 0.18	± 0.05	± 0.16
	98%	96%	100%	50%//50%	99%	100%	96%	100%	87%	100%	100%	100%
*MKL2*	0.64	0.66	0.62	1.24	0.64	0.68	1.27	0.63	0.63	0.59	0.63	0.63
NM_014048	± 0.17	± 0.17	± 0.16	± 0.11	± 0.17	± 0.15	± 0.17	± 0.16	± 0.15	± 0.18	± 0.15	± 0.16
	73%	70%	81%	100%	74%	92%	64%	90%	81%	100%	81%	90%
*CLOCK*	0.52	0.53	0.51	0.86	0.50	0.60	1.19	0.48	0.68	0.51	0.35	0.52
NM_004898	± 0.2	± 0.2	± 0.21	± 0.18	± 0.20	± 0.13	± 0.20	± 0.19	± 0.18	± 0.17	± 0.10	± 0.21
	77%	77%	78%	100%	76%	77%	79%	73%	80%	78%	100%	73%
*KIA1576*	0.06	0.06	0.05	0.3	0.06	0.03	1.04	0.05	0.07	0.07	0.07	0.05
NM_020927	± 0.08	± 0.54	± 0.07	± 0.17	± 0.09	± 0.02	± 0.09	± 0.08	± 0.06	± 0.11	± 0.10	± 0.06
	97%	93%	100%	100%	98%	92%	89%	97%	93%	100%	100%	97%
*C10orf32*	0.60	0.59	0.62	0.83//1.23	0.59	0.69	1.26	0.6	0.76	0.55	0.47	0.63
NM_144591	± 0.20	± 0.21	± 0.19		0.20	0.20	0.21	0.19	0.18	0.18	0.18	0.19
	80%	75%	89%	50%//50%	79%	92%	75%	90%	67%	74%	75%	89%
*CLMN*	0.48	0.50	0.44	0.60//1.30	0.51	0.37	1.26	0.49	0.54	0.54	0.45	0.46
NM_024734	± 0.19	± 0.19	± 0.18		± 0.19	± 0.16	± 0.19	± 0.19	± 0.13	± 0.21	± 0.28	± 0.16
	89%	89%	89%	50%//50%	88%	100%	85%	97%	70%	96%	100%	89%
*CAPN2*	0.68	0.69	0.66	1.42	0.68	0.66	1.23	0.65	0.72	0.72	0.61	0.67
NM_001748	± 0.16	± 0.16	± 0.14	± 0.16	± 0.15	± 0.17	± 0.16	± 0.14	± 0.15	± 0.19	± 0.15	± 0.14
	71%	70%	74%	100%	74%	72%	68%	77%	60%	70%	100%	70%
*AMFR*	0.53	0.52	0.55	0.44	0.53	0.56	1.2	0.51	0.55	0.55	0.43	0.56
NM_001144	± 0.16	± 0.16	± 0.18	± 0.03	± 0.17	± 0.13	± 0.18	± 0.13	± 0.18	± 0.16	± 0.08	± 0.17
	93%	95%	89%	100%	94%	92%	91%	97%	100%	91%	100%	89%
*ST8SIA2*	0.54	0.52	0.59	0.8//1.31	0.53	0.6	1.60	0.58	0.55	0.49	0.66	0.55
NM_006011	± 0.21	± 0.22	± 0.18		± 0.21	± 0.18	± 0.21	± 0.19	± 0.21	± 0.23	± 0.22	± 0.18
	72%	73%	70%	50%//50%	74%	72%	70%	79%	70%	70%	100%	78%

± standard deviation; % percent of samples in group; *T* T-status, *G* grading, *N* lymph nodes, *UICC* UICC classification

^a^group G1 includes two samples, the results are less robust;//both results in group G1 are shown

**Table 4:** Expression fold changes of putative target genes for miR-31 that are not yet precisely assignable genes

**Gene**	**All**	**T1 + T2**	**T3 + T4**	**G1**^**a**^	**G2**	**G3**	**N0**	**N + **	**UICC 1**	**UICC 2**	**UICC 3**	**UICC 4**
	*n* = 83	*n* = 56	*n* = 27	*n* = 2	*n* = 68	*n* = 13	*n* = 53	*n* = 30	*n* = 16	*n* = 23	*n* = 8	*n* = 36
hsa-miR-31	10.77 ± 11.96 80%	10.08 ± 11.83 80%	12.39 ± 12.29 81%	0.36 ± 0.12 100%	10.13 ± 12.16 81%	14.12 ± 10.81 92%	10.45 ± 12.33 85%	11.66 ± 11.4 72%	6.43 ± 5.98 70%	11.12 ± 14.43 95%	9.09 ± 10.71 75%	12.69 ± 11.97 78%
*ERBB3*	0.45	0.43	0.48	0.51//1.48	0.45	0.47	1.78	0.41	0.51	0.45	0.27	0.41
NM_001982	± 0.23	± 0.26	± 0.18		± 0.23	± 0.26	± 0.22	± 0.25	± 0.27	± 0.23	± 0.22	± 0.25
	92%	91%	93%	50%//50%	94%	85%	92%	90%	93%	87%	100%	90%
*ALG5*	0.63	0.61	0.66	0.8//1.95	0.63	0.65	1.35	0.59	0.67	0.65	0.61	0.63
NM_013338	± 0.17	± 0.17	± 0.17		± 0.18	± 0.16	± 0.17	± 0.17	± 0.16	± 0.18	± 0.17	± 0.18
	81%	80%	81%	50%//50%	79%	92%	77%	87%	67%	87%	100%	78%
*ZBTB10*	0.41	0.41	0.41	0.70//1.59	0.41	0.39	1.49	0.45	0.44	0.37	0.48	0.4
NM_023929	± 0.15	± 0.15	± 0.15		± 0.15	± 0.14	± 0.13	± 0.17	± 0.18	± 0.11	± 0.09	± 0.17
	83%	80%	89%	50%//50%	82%	92%	81%	87%	70%	83%	100%	84%
*TNRC6B*	0.64	0.65	0.62	0.81	0.63	0.74	1.27	0.67	0.75	0.59	0.57	0.66
NM_015088	± 0.2	± 0.19	± 0.20	± 0.01	± 0.2	± 0.14	± 0.21	± 0.18	± 0.13	± 0.2	± 0.25	± 0.19
	82%	82%	81%	100%	85%	62%	85%	77%	87%	83%	100%	76%
*ERBB2IP*	0.74	0.75	0.73	0.92//1.02	0.74	1.12	1.11	0.75	0.81	0.72	0.74	0.76
NM_018695	± 0.15	± 0.15	± 0.16		± 0.15	± 0.16	± 0.15	± 0.16	± 0.13	± 0.14	± 0.21	± 0.14
	70%	73%	63%	50%//50%	75%	54%	72%	67%	70%	74%	100%	62%
*RAB3C*	0.29	0.34	0.21	0.08//1.17	0.33	0.14	2.11	0.31	0.50	0.28	0.28	0.26
NM_138453	± 0.24	± 0.26	± 0.17		± 0.24	± 0.21	± 0.26	± 0.22	± 0.33	± 0.22	± 0.21	± 0.21
	88%	84%	96%	50%//50%	87%	100%	87%	90%	70%	87%	100%	95%
*RAB27B*	0.54	0.51	0.60	0.39//0.97	0.54	1.17	1.36	0.54	0.5	0.54	0.57	0.55
NM_004163	± 0.26	± 0.27	± 0.23		± 0.26	± 0.16	± 0.27	± 0.27	± 0.31	± 0.24	± 0.26	± 0.27
	70%	75%	59%	50%//50%	75%	54%	68%	73%	70%	65%	100%	70%
*CXCL12*	0.31	0.34	0.27	0.41	0.34	0.2	1.35	0.33	0.44	0.28	0.39	0.29
NM_000609	± 0.21	± 0.23	± 0.17	± 0.17	± 0.22	± 0.16	± 0.22	± 0.20	± 0.3	± 0.17	± 0.25	± 0.19
	86%	80%	96%	100%	85%	92%	85%	87%	70%	83%	88%	92%
*VAV3*	0.46	0.43	0.52	0.36	0.16	0.44	1.18	0.44	0.35	0.52	0.38	0.48
NM_006113	± 0.23	± 0.24	± 0.19	± 0.21	± 0.13	± 0.32	± 0.22	± 0.25	± 0.21	± 0.22	± 0.21	± 0.24
	81%	82%	85%	100%	100%	72%	81%	83%	87%	78%	88%	78%

± standard deviation; % percent of samples in group; *T* T-status, *G* grading, *N* lymph nodes, *UICC* UICC classification

^a^group G1 includes two samples, the results are less robust;//both results in group G1 are shown)

Based on our literature research, we subdivided the putative target genes into known tumour suppressor genes, known oncogenes, and genes that have not yet been fully characterized. We were able to isolate 20 potential target genes. Gene expression significantly correlated with the expression of miR-31. Our studies identified the following known tumour suppressor genes listed in Table 2 as putative target genes for miR-31. Correlations with various OSCC parameters and gene expression levels were analysed statistically, with a p value of 0.05. In some cases, the p-values slightly exceeded the enabled threshold of 0.05.

The PCDH9 gene was significantly associated with tumour grade (*p* = 0.068) and smoking status (*p* = 0.003). Previous research has shown a link between *PCDH9*, an oncogenic or suppressor factor, and tumour development in other tumour types [16]. In 2022, another research group also found exactly this correlation in HNSCC, which confirms our results [17]. With increasing tumour size, differentiation, metastasis and lymph node involvement, the expression of *PCDH9* decreased in our samples. Gene expression is significantly decreased in smokers, which could explain the increased risk of OSCC in smokers. In conclusion, the expression of *PCDH9* might serve as a potential prognostic marker, with decreasing *PCDH9* expression indicating a worse prognosis. *MEIS* is known as a potential tumour suppressor and has already been identified as a central driver in several human leukaemia types. However, *MEIS* family expression data are highly variable across solid tumour types and can also vary within a single tumour type [18]. In our samples, *MEIS* was significantly associated with UICC status (*p* = 0.018), grade (*p* = 0.002) and type (*p* = 0.063). Since *MEIS* expression is variable in different tissues, more data should be obtained on this gene to confidently promote its use as a prognostic marker. Since *MEIS* is required for haematopoiesis, megakaryocyte lineage development and vascular patterning, among other factors, *MEIS* could be classified as a potentially important angiogenesis factor in OSCC [18–20]. In our samples, *CNOT4* expression was significantly associated with tumour grade (*p* = 0.06) and type (*p* = 0.002). Another research group has shown that increased expression of *CNOT4* in the intestinal mucosa is associated with an increased risk of colon cancer [21]. Further research should be conducted to determine to what extent *CNOT4* might also be a relevant target gene in the mucosa of the head and neck region. It has so far only been investigated in other tumour types, particularly in lung cancer [22]. *GRHL1* expression was related to N status (*p* = 0.048) and smoking status (*p* = 0.028). *GRHL1* encodes a transcription factor that plays an important role in inhibiting the growth, proliferation, and progression of embedded tumour cells. A publication investigating the relationship between *GRHL1* expression and the prognostic value of *GRHL1* in squamous cell carcinomas of the oesophagus showed that low expression of *GRHL1* was associated with poor differentiation and a lower overall survival rate [23]. *GRHL1* was downregulated in 84% of our samples. However, no significant correlation between expression levels and differentiation could be established. In contrast, research on endometrial cancer has already established a link between expression and the overall survival rate. High expression was associated with a higher mortality rate [24]. The correlation between *GRHL1* expression and metastasis formation in relation to N status should be further investigated. *NFIA* expression significantly correlated with UICC (*p* = 0.035) and T-status (*p* = 0.019). It has already been established that lower *NFIA* expression is associated with a lower overall survival rate in patients with head and neck tumours [25]. In addition, another study on *NFIA* expression in oesophageal SCC patients revealed a significant correlation between *NFIA* expression and the degree of cell differentiation, TNM classification, N status and overall survival. Consequently, it was shown that high *NFIA* expression is an independent prognostic factor for oesophageal SCC [26]. *PLCB1* expression was significantly related to T stage (*p* = 0.034), tumour grade (*p* = 0.068) and the type of OSCC (*p* = 0.007). *PLCB1* has been previously shown to be an important tumour suppressor gene in OSCC and is often downregulated in these tumours [27]. According to our data, *PLCB1* was downregulated in 77% of the samples. *PLCBI* is also described as an oncogene [28]. *DIRAS* was downregulated in 89% of all the samples. It correlated significantly with UICC status (*p* = 0.088), alcohol abuse (*p* = 0.046), and nicotine consumption (*p* = 0.067). It has already been established in other studies that *DIRAS3* serves as a significant tumour suppressor in head and neck SCC and is often silenced [29, 30]. In particular, the relationship between nicotine and alcohol consumption in terms of reduced expression of *DIRAS* could be another explanation for the increased risk associated with elevated nicotine and alcohol intake. *ALDH5A1* was downregulated in 79% of the OSCC samples. The expression data correlated significantly with the UICC status (*p* = 0.026) and T status (*p* = 0.01). The aldehyde-dehydrogenase-5-family belongs to the superfamily of aldehyde dehydrogenases (ALDHs) and is already used in other tumours as a marker for patient prognosis [31]. Furthermore, a correlation between *ALDH5A1* and response to chemoradiotherapy was identified in oesophageal squamous cell carcinomas. In a model of different genes, the response to therapy was dependent on the expression of the selected 10 genes [32]. *SPTBN-1* was significantly associated with UICC status (*p* = 0.047), T status (*p* = 0.088) and epithelial type (*p* = 0.038) in our OSCC samples. It was downregulated in 98% of the samples. *SPTBN-1* is frequently mutated in HNSCC, HPV + , and OPSCCs [33]. It has already been found to serve as a prognostic factor in hepatocellular carcinoma, oesophageal SCC and pancreatic carcinoma [34]. *DLC1* was downregulated in 75% of the OSCC samples. It correlated significantly with T-status (*p* = 0.028), smoking status (*p* = 0.057), alcohol abuse status (*p* = 0.04) and epithelial type of OSCC (*p* = 0.01). In nasopharyngeal carcinoma, ectopic expression of *DLC1* led to a decrease in colony formation and invasion [35]. It has been identified as a promising gene for patient prognosis in OSCC [36].

Our study identified 25 genes, which are known oncogenes, as putative target genes of miR-31 (Table 3). Correlations with various OSCC parameters and gene expression levels were analysed statistically, with a p value of 0.05.

The selenoprotein *SELK* was downregulated in 93% of the samples, and the expression of *SELK* showed a significant correlation with UICC status (*p* = 0.022), T status (*p* = 0.036) and tumour grade (*p* = 0.073). Studies have described the relationship between microRNAs and selenoproteins in other tumour types. In hepatocellular carcinoma, there was a significant association between miR-544a and *SELK* expression. An important approach to treating this type of tumour has also been described [37]. Selenoproteins transport the essential trace minerals selenium. This activity is significant for glutathione peroxidase, which protects the cell from oxidative stress and the formation of radicals. There was a significant correlation between the expression of *C1QTNF7* and UICC status (*p* = 0.04), T status (*p* = 0.00028), smoking status (*p* = 0.028), alcohol abuse status (*p* = 0.033), and epithelial type of OSCC (*p* = 0.044). The gene was downregulated in 98% of the samples. To date, little is known about *C1QTNF7* gene expression in OSCC. In 2020, *C1QTNF7* was identified as a critical prognostic marker in ovarian cancer that was significantly related to overall patient survival [38]. *C1QTNF7* was found to be downregulated after the first 2 Gy fraction of radiotherapy in patients with tumours in the head and neck region [39]. *NEBL* was correlated with tumour grade (*p* = 0.036) and smoking status (*p* = 0.073), and its expression was downregulated in 94% of patients. *NEBL* is significantly overexpressed in most cases of colorectal cancer (CRC). Here, an association between *PD-L1* expression and lymph node metastasis was established. Therefore, *NEBL* is considered a prognostic marker in CRC [40]. In relation to our results, research should also be conducted on *NEBL* as a prognostic marker in OSCC. *PDE5A* was downregulated in 95% of the OSCC samples and correlated with T-status (*p* = 0.033), smoking status (*p* = 0.076), alcohol abuse status (*p* = 0.015), and epithelial type (*p* = 0.029). Phosphodiesterase influences the degradation of cyclic nucleotides. Phosphodiesterases are already focussed on tumour research. The expression of *PDE5A* was increased in colorectal neoplasms [41] and in 2021, a meta-analysis about their effect on the risk of melanoma was investigated [42]. *EPM2AIP1* expression correlated with UICC status (*p* = 0.048), grade (*p* = 0.089), smoking status (*p* = 0.036), alcohol abuse status (*p* = 0.038), and epithelial type of OSCC (*p* = 0.073). *EPM1AIP1* was downregulated in 88% of the OSCC samples. In a study on sessile serrated adenomas (SSAs), the expression of *EPM2AIP1* was frequently decreased compared to that in colorectal carcinomas. SSAs give rise to 20–30% of colorectal carcinomas [43]. *MKL2* expression was significantly related to UICC status (*p* = 0.014), T status (*p* = 0.05), N status (*p* = 0.05), and epithelial type of OSCC (*p* = 0.02). The gene was downregulated in 83% of the samples. In another study, miR-532-5p was shown to have a tumour-promoting effect on lung adenocarcinoma by targeting *MKL2* [44]. *CLMN* was downregulated in 89% of the OSCC samples. The expression correlated with tumour grade (*p* = 0.017). In HNSCC, *CLMC* frequently appears to be mutated [45]. In general, very little research has been done in this context about *CLMC* and cancer.

Our studies identified nine genes as putative target genes of miR-31, but these genes have not yet been definitively characterized (Table 4). Correlations with various OSCC parameters and gene expression levels were analysed statistically, with a *p* value of 0.05. In some cases, the p values slightly exceeded the threshold of 0.05.

*ERBB3* was related to tumour grade (*p* = 0.078) and smoking status (*p* = 0.003) and was downregulated in 92% of the patients. *ERBB3* has already been identified as a promoter of tumours in the head and neck region [46]. In addition, *ERBB3* has also been identified as a possible target gene for targeted tumour therapy [47]. Our data strongly suggest that special attention should be given to this gene for tumour therapy. *RAB3C* expression correlated with UICC status (*p* = 0.051), grade (*p* = 0.023) and T status (*p* = 0.015) and was downregulated in 88% of the samples. In other tumour types, such as colorectal cancer, overexpression of *RAB3C* was associated with poor prognosis, including increased metastasis [48]. *VAV3* expression correlated with tumour grade (*p* = 0.069) and smoking status (*p* = 0.055) in our cohort. *VAV3* was downregulated in 81% of the OSCC samples. Previous studies have shown that different subtypes of *VAV3* are related to the tumour aggressiveness of OSCC and are frequently downregulated [49]. Our results also indicate this connection.

## Conclusion

The aim of this study was to determine the expression patterns of target genes of human miR-31 in 83 OSCC samples, as well as their correlation with tumour parameters. Thus, we try to bring prognostic factors in connection with target genes. A literature search identified the target genes. This summary should help to delimit the vast number of possible target genes computed to a functional group. In doing so, we not only considered and compared known genes in SCC, but also identified new potential target genes. Previously known genes involved in head and neck tumours include *MEIS*, *PLCB1*, *DIRAS*, *DLC1*, *VAV3*, *C1QTNF7*, and *CLMN*. Through our investigations, we were able to confirm the importance of these genes, which should serve as a basis for further research and recognizing predictive factors. This could ultimately improve the diagnosis, prognosis, and treatment of patients with OSCC and consequently also optimize their usually poor long-term prognosis. Therefore, further investigations based on our results are essential. In future studies, our results provide a basis for expression data and excellent starting points that should further clarify the pathways affected by AngiomiRs.

## Data Availability

No datasets were generated or analysed during the current study.

## Abbreviations

OSCC/Oral SCC: Oral squamous cell carcinoma
SCC: Squamous cell carcinoma
HNSCC: Head and neck squamous cell carcinoma
AngiomiRs/AngiomiRNAs: AngiomicroRNAs
miR: MicroRNA
RNA: Ribonucleic acid
TNM: Tumour, node, metastasis
UTR: Untranslated region
UICC: Union internationale Contre le Cancer
RT‒qPCR: Real-time quantitative real-time PCR
PCR: Polymerase chain reaction

## References

[1] Markopoulos, AK. Current aspects on oral squamous cell carcinoma. Open Dent J. 2012.10.2174/1874210601206010126. 10.2174/1874210601206010126 PMC3428647 22930665

[2] Kim J, Yao F, Xiao Z, Sun Y, Ma L. MicroRNAs and metastasis: small RNAs play big roles. Cancer Metastasis Rev. 2018.10.1007/s10555-017-9712-y. 10.1007/s10555-017-9712-y PMC5803344 29234933

[3] Hashemi A, Gorji-Bahri G. MicroRNA: promising roles in cancer therapy. Curr Pharm Biotechnol. 2020.10.2174/1389201021666200420101613. 10.2174/1389201021666200420101613 32310047

[4] Annese T, Tamma R, De Giorgis M, Ribatti D. microRNAs biogenesis, functions and role in tumor angiogenesis. Front Oncol. 2020.10.3389/fonc.2020.581007. 10.3389/fonc.2020.581007 PMC7729128 33330058

[5] Li M, Dong Y, Chen Z, Meng L, Liu X, Zhang X, Wang H, et al. MicroRNA-31 negatively regulates interleukin-34 expression in vitro. Immunol Invest. 2019.10.1080/08820139.2019.1578230. 10.1080/08820139.2019.1578230 31012336

[6] Suárez Y, Wang C, Manes TD, Pober JS. Cutting edge: TNF-induced microRNAs regulate TNF-induced expression of E-selectin and intercellular adhesion molecule-1 on human endothelial cells: feedback control of inflammation. J Immunol. 2010.10.4049/jimmunol.0902369. 10.4049/jimmunol.0902369 PMC2797568 19949084

[7] Ribatti D, Tamma R. Epigenetic control of tumor angiogenesis. Microcirculation. 2020.10.1111/micc.12602. 10.1111/micc.12602 31863494

[8] Jung S, Sielker S, Purcz N, Sproll C, Raem A, Kleinheinz J. expression profile of angiogenesis related genes in head and neck squamous cell carcinoma. J Biochip Tissue Chip. 2013.10.4172/2153-0777.1000104.

[9] Li J, Huang H, Sun L, Yang M, Pan C, Chen W, Wu D, et al. MiR-21 indicates poor prognosis in tongue squamous cell carcinomas as an apoptosis inhibitor. Clin Cancer Res. 2009.10.1158/1078-0432.CCR-08-3053. 10.1158/1078-0432.CCR-08-3053 19509158

[10] Dioguardi M, Spirito F, Sovereto D, Alovisi M, Troiano G, Aiuto R, Garcovich D, et al. MicroRNA-21 expression as a prognostic biomarker in oral cancer: systematic review and meta-analysis. Int J Environ Res Public Health. 2022.10.3390/ijerph19063396. 10.3390/ijerph19063396 PMC8948874 35329083

[11] Deng HT, Liu HL, Zhai BB, Zhang K, Xu GC, Peng XM, Zhang QZ, Li LY. Vascular endothelial growth factor suppresses TNFSF15 production in endothelial cells by stimulating miR-31 and miR-20a expression via activation of Akt and Erk signals. FEBS Open Bio. 2016.10.1002/2211-5463.12171. 10.1002/2211-5463.12171 PMC5221472 28097093

[12] Jung S, Sielker S, Purcz N, Sproll C, Açil Y, Kleinheinz J. Analysis of angiogenic markers in oral squamous cell carcinoma-gene and protein expression. Head Face Med. 2015. 10.1186/s13005-015-0076-7. 10.1186/s13005-015-0076-7 PMC4461981 26044849

[13] Safari Z, Firouzi A, Rezaeikalantari N, Mohammadi S, Ranjbar N, Shahpori H, Khaleghi P, et al. The salivary exosomal microRNA as a potential biomarker in patients with periodontitis and oral cancers. Chem Biol Drug Des. 2023.10.1111/cbdd.14159. 10.1111/cbdd.14159 36301416

[14] Rodrigues-Junior DM, Tan SS, Lim SK, de Souza Viana L, Carvalho AL, Vettore AL, Iyer NG. High expression of MLANA in the plasma of patients with head and neck squamous cell carcinoma as a predictor of tumor progression. Head Neck. 2019.10.1002/hed.25510. 10.1002/hed.25510 30803092

[15] Desel I, Jung S, Purcz N, Açil Y, Sproll C, Kleinheinz J, Sielker S. Analysis of genes related to invadopodia formation and CTTN in oral squamous cell carcinoma—A systematic gene expression analysis. Curr Issues Mol.Biol. 2023.10.3390/cimb45080437. 10.3390/cimb45080437 PMC10453299 37623256

[16] Izycka N, Sterzynska K, Januchowski R, Nowak-Markwitz E. Semaphorin 3A (SEMA3A), protocadherin 9 (PCdh9), and S100 calcium binding protein A3 (S100A3) as potential biomarkers of carcinogenesis and chemoresistance of different neoplasms, including ovarian cancer - review of literature. Ginekol Pol. 2019.10.5603/GP.2019.0040. 10.5603/GP.2019.0040 31059116

[17] Liu S, Liu W, Ding, Z, Yang X, Jiang Y, Wu Y, Liu Y, Wu J. Identification and validation of a novel tumor driver gene signature for diagnosis and prognosis of head and neck squamous cell carcinoma. Front Mol Biosci. 2022. 10.3389/fmolb.2022.912620. 10.3389/fmolb.2022.912620 PMC9631213 36339718

[18] Schulte D, Geerts D. MEIS transcription factors in development and disease. Development. 2019.10.1242/dev.174706. 10.1242/dev.174706 31416930

[19] Paul S, Zhang X, He JQ. Homeobox gene Meis1 modulates cardiovascular regeneration. Semin Cell Dev Biol. 2020.10.1016/j.semcdb.2019.10.003. 10.1016/j.semcdb.2019.10.003 PMC7839269 31623926

[20] MEIS1 Gene (Protein Coding). GeneCards GC02P066433. Accessed 02 Nov 2023.

[21] Tsuda M, Noguchi M, Kurai T, Ichihashi Y, Ise K, Wang L, Ishida Y, et al. Aberrant expression of MYD88 via RNA-controlling CNOT4 and EXOSC3 in colonic mucosa impacts generation of colonic cancer. Cancer Sci. 2021.10.1111/cas.15157. 10.1111/cas.15157 PMC8645755 34626022

[22] Zhang B, Zhao B, Han S, Chen S. CNOT4 suppresses nonsmall cell lung cancer progression by promoting the degradation of PAF1. Mol Carcinog. 2023.10.1002/mc.23599. 10.1002/mc.23599 37493105

[23] Li M, Li Z, Guan X, Qin Y. Suppressor gene GRHL1 is associated with prognosis in patients with oesophageal squamous cell carcinoma. Oncol Lett. 2019.10.3892/ol.2019.10072. 10.3892/ol.2019.10072 PMC6444427 30944626

[24] Guo S, Bai W, Cui F, Chen X, Fang X, Shen H, Gu X. Exploration of the correlation between GRHL1expression and tumor microenvironment in endometrial cancer and immunotherapy. Pharmgenomics Pers Med. 2024.10.2147/PGPM.S453061. 10.2147/PGPM.S453061 PMC10999208 38586176

[25] Li Y, Sun C, Tan Y, Li L, Zhang H, Liang Y, Zeng J, Zou H. Transcription levels and prognostic significance of the NFI family members in human cancers. PeerJ. 2020. 10.7717/peerj.8816. 10.7717/peerj.8816 PMC7085295 32219034

[26] Yang B, Zhou ZH, Chen L, Cui X, Hou JY, Fan KJ, Han SH, Li P, et al. Prognostic significance of NFIA and NFIB in esophageal squamous carcinoma and esophagogastric junction adenocarcinoma. Cancer Med. 2018. 10.1002/cam4.1434. 10.1002/cam4.1434 PMC5943462 29577671

[27] Guerrero-Preston R, Michailidi C, Marchionni L, Pickering CR, Frederick MJ, Myers JN, Yegnasubramanian S, et al. Key tumor suppressor genes inactivated by "greater promoter" methylation and somatic mutations in head and neck cancer. Epigenetics. 2014.10.4161/epi.29025. 10.4161/epi.29025 PMC4143405 24786473

[28] Guerrero-Preston R, Michailidi C, Marchionni L, Pickering CR, Frederick MJ, Myers JN, Yegnasubramanian S, et al. Key tumor suppressor genes inactivated by "greater promoter" methylation and somatic mutations in head and neck cancer. Epigenetics. 2014.10.1186/2043-9113-1-21. 10.4161/epi.29025 PMC4143405 24786473

[29] Zhang S, Feng XL, Shi L, Gong CJ, He ZJ, Wu HJ, Ling TY. Genome-wide analysis of DNA methylation in tongue squamous cell carcinoma. Oncol Rep. 2013.10.3892/or.2013.2309. 10.3892/or.2013.2309 23446731

[30] Liu Z, Hurst DR, Qu X, Lu LG, Wu CZ, Li YY, Li Y. Reexpression of DIRAS3 and p53 induces apoptosis and impaired autophagy in head and neck squamous cell carcinoma. Mil Med Res. 2020.10.1186/s40779-020-00275-3. 10.1186/s40779-020-00275-3 PMC7548045 33038921

[31] Tian X, Han Y, Yu L, Luo B, Hu Z, Li X, Yang Z, et al. Decreased expression of ALDH5A1 predicts prognosis in patients with ovarian cancer. Cancer Biol Ther. 2017.10.1080/15384047.2017.1295175. 10.1080/15384047.2017.1295175 PMC5450734 28346042

[32] Song K, Gu B, Ma C, Wang B, Wang N, Yu R, Chen H. Epithelial-mesenchymal transi-tion gene signature is associated with neoadjuvant chemoradiotherapy resistance and prognosis of esophageal squamous Cell Carcinoma. Dis Markers. 2022.10.1155/2022/3534433. 10.1155/2022/3534433 PMC9442501 36072903

[33] Grønhøj C, Jensen DH, Agander T, Kiss K, Høgdall E, Specht L, Bagger FO, et al. Deep sequencing of human papillomavirus positive loco-regionally advanced oropharyngeal squamous cell carcinomas reveals novel mutational signature. BMC Cancer. 2018.10.1186/s12885-018-4567-3. 10.1186/s12885-018-4567-3 PMC5992702 29879932

[34] Wang SY, Feng LY, Meng ZQ. Bicluster and pathway enrichment analysis related to tumor progression of hepatocellular carcinoma. Eur Rev Med Pharmacol Sci. 2015;19:1191–7. 25912578

[35] Loyo M, Brait M, Kim MS, Ostrow KL, Jie CC, Chuang AY, Califano JA, et al. A survey of methylated candidate tumor suppressor genes in nasopharyngeal carcinoma. Int J Cancer. 2011.10.1002/ijc.25443. 10.1002/ijc.25443 PMC2955155 20473931

[36] Pratama R, Hwang JJ, Lee JH, Song G, Park HR. Authentication of differential gene ex-pression in oral squamous cell carcinoma using machine learning applications. BMC Oral Health. 2021.10.1186/s12903-021-01642-9. 10.1186/s12903-021-01642-9 PMC8164276 34051764

[37] Potenza N, Castiello F, Panella M, Colonna G, Ciliberto G, Russo A, Costantini S. Human MiR-544a modulates SELK expression in hepatocarcinoma cell lines. PLoS One. 2016.10.1371/journal.pone.0156908. 10.1371/journal.pone.0156908 PMC4898719 27275761

[38] An Y, Yang Q. Development and validation of an immune-related prognostic signature for ovarian cancer based on weighted gene coexpression network analysis. Biomed Res Int. 2020.10.1155/2020/7594098. 10.1155/2020/7594098 PMC7749778 33381581

[39] Nie YH, Liu XD, Huang R, Xie DF, Yin WJ, Guan H, Yu ZJ, Zhou PK. Analysis of mRNA expression patterns in peripheral blood cells of 3 patients with cancer after the first fraction of 2 Gy irradiation: an integrated case report and systematic review. Dose Response. 2019.10.1177/1559325819833474. 10.1177/1559325819833474 PMC6393837 30833875

[40] Hosseini SM, Mahjoubi F, Majidzadeh T, Khaje-Hosseini F, Haghipanah M. Nebulette expression is associated with lymph node metastasis in patients with colorectal cancer. Middle East J Dig Dis. 2018.10.15171/mejdd.2018.107. 10.15171/mejdd.2018.107 PMC6119834 30186581

[41] Mahmood B, Damm MM, Jensen TS, Backe MB, Dahllöf MS, Poulsen SS, Bindslev N, Hansen MB. Phosphodiesterases in nonneoplastic appearing colonic mucosa from patients with colorectal neoplasia. BMC Cancer. 2016.10.1186/s12885-016-2980-z. 10.1186/s12885-016-2980-z PMC5141637 27927168

[42] Lu YP, Fan S, Liang Z, Song Y, Liu K, Zhou K, Wang X, Kang J, Yang Y, Liu X. Phos-phodiesterase type 5 inhibitors and risk of skin cancers in men: a meta-analysis and trial sequential analysis involving 7,479,852 subjects. World J Mens Health. 2021.10.5534/wjmh.200082. 10.5534/wjmh.200082 PMC8443994 33151043

[43] Liu C, Fennell LJ, Bettington ML, Walker NI, Dwine J, Leggett BA, Whitehall VLJ. DNA methylation changes that precede onset of dysplasia in advanced sessile serrated adenomas. Clin Epigenetics. 2019.10.1186/s13148-019-0691-4. 10.1186/s13148-019-0691-4 PMC6570920 31200767

[44] Griesing S, Kajino T, Tai MC, Liu Z, Nakatochi M, Shimada Y, Suzuki M, Takahashi T. Thyroid transcription factor-1-regulated microRNA-532-5p targets KRAS and MKL2 oncogenes and induces apoptosis in lung adenocarcinoma cells. Cancer Sci. 2017.10.1111/cas.13271. 10.1111/cas.13271 PMC5497805 28474808

[45] Ghias K, Rehmani SS, Razzak SA, Madhani S, Azim MK, Ahmed R, Khan MJ. Mutational landscape of head and neck squamous cell carcinomas in a South Asian population. Genet Mol Biol. 2019.10.1590/1678-4685-GMB-2018-0005. 10.1590/1678-4685-GMB-2018-0005 PMC6905448 31188922

[46] Alvarado D, Ligon GF, Lillquist JS, Seibel SB, Wallweber G, Neumeister VM, Rimm DL, et al. ErbB activation signatures as potential biomarkers for anti-ErbB3 treatment in HNSCC. PLoS One. 2017.10.1371/journal.pone.0181356. 10.1371/journal.pone.0181356 PMC5517012 28723928

[47] Erjala K, Sundvall M, Junttila TT, Zhang N, Savisalo M, Mali P, Kulmala J, Pulkkinen J, et al. Signalling via ErbB2 and ErbB3 associates with resistance and epidermal growth factor receptor (EGFR) amplification with sensitivity to EGFR inhibitor gefitinib in head and neck squamous cell carcinoma cells. Clin Cancer Res. 2006.10.1158/1078-0432.CCR-05-2404. 10.1158/1078-0432.CCR-05-2404 16818711

[48] Chang YC, Su CY, Chen MH, Chen WS, Chen CL, Hsiao M. Secretory RAB GTPase 3C modulates IL6-STAT3 pathway to promote colon cancer metastasis and is associated with poor prognosis. Mol Cancer. 2017.10.1186/s12943-017-0687-7. 10.1186/s12943-017-0687-7 PMC5547507 28784136

[49] Trenkle T, Hakim SG, Jacobsen HC, Sieg P. Differential gene expression of the proto-oncogene VAV3 and the transcript variant VAV31 in oral squamous cell carcinoma. Anticancer Res. 2015;35:2593–600. 25964534

